# Analysis of Clinical Factors Associated with Medical Burden and Functional Status in Pyogenic Spine Infection

**DOI:** 10.3390/jcm12072551

**Published:** 2023-03-28

**Authors:** Seongmin Jeon, Dongwoo Yu, Sang Woon Bae, Sang Woo Kim, Ikchan Jeon

**Affiliations:** 1 Department of Neurosurgery, College of Medicine, Yeungnam University, Daegu 42415, Republic of Korea; yee4374@naver.com (S.J.); icarus0710@hanmail.net (D.Y.); sw902@ynu.ac.kr (S.W.K.); 2 Department of Infectious Medicine, College of Medicine, Yeungnam University, Daegu 42415, Republic of Korea; sangoon@gmail.com

**Keywords:** pyogenic, spine infection, vertebral osteomyelitis, burden, disability

## Abstract

Background and purpose: Pyogenic spinal infection (PSI) has recently been on the rise due to aging and increasing degenerative spinal disease related procedures. PSI requires long-term antibiotic treatment and is followed by sustained functional disability even after successful treatment. This study aimed to analyze the clinical factors associated with medical burden and functional status of PSI. Methods: This retrospective study involved patients with non-postoperative PSI of thoraco-lumbo-sacral area in a single tertiary hospital. The length/cost of hospitalization with an antibiotic therapy and severity of back pain using the short form 36 (SF-36) were defined as the medical burden and functional status, respectively. We analyzed the clinical factors associated with medical burden and functional status. Results: We enrolled 142 patients (91 males and 51 females). The length and cost of hospitalization were 55.56 ± 27.09 (7–172) days and $14,070.17 ± 9289.39 (1611.87–48,722.35), respectively. A recurrence rate of 7.7% (11/142) and significant improvement of SF-36 at six months after completion of antibiotic treatment were noted (*p* < 0.05). Procedure-related (OR 2.702), C-reactive protein (CRP; OR 1.062), bacteremia (OR 4.966), additional surgical treatment (OR 6.524), recurrence (OR 12.453), and paraspinal abscess (OR 5.965) for above-average length of hospitalization were observed; female (OR 4.438), CRP (OR 1.071), bacteremia (OR 4.647), additional surgical treatment (OR 6.737), recurrence (OR 22.543), and extent of lesion (OR 1.431) for above-average cost of hospitalization; leg weakness (OR 15.966), white blood cell (WBC; OR 1.116), Charlson’s comorbidity index (CCI, OR 1.485), and identification of causative bacteria (OR 2.913) for below-average initial SF-36 were observed; leg weakness (OR 7.975) and WBC (OR 1.094) for below-average 6-month SF-36 were the statistically significant clinical factors in the multivariable logistic regression analysis (*p* < 0.05). Conclusion: Recurrence and leg weakness were identified as the most important clinical factors for medical burden and functional status in PSI, respectively. We think that it is necessary to actively suppress recurrence and manage neurological deficits for decreasing medical burden and achieving favorable functional outcome in the treatment of PSI.

## 1. Introduction

Spine infection is an infectious disease which can be developed by pyogenic, tuberculous, or brucellar causes, with pyogenic origin being the most common cause. The incidence of pyogenic spine infection (PSI) is increasing due to an aging population with degenerative spine diseases, chronic immune-compromising diseases, frequent spine procedures, and diagnostic advancement [1,2]. The annual incidence of hospitalization with PSI in the United States between 1998 and 2013 rose from 2.9 to 5.4 per 100,000 individuals [3]. Among the causes of PSI, spine procedures such as spine surgery, epidural injection, nerve root block, or discography are arising as the primary routes of PSI through hematogenous spread or direct inoculation of virulent organisms with an increasing prevalence of degenerative spine diseases [4].

There is still no clear guidance regarding the duration and administration route for antibiotics in the treatment of PSI. Generally, an extended course of parenteral antibiotics followed by a maintenance course of oral antibiotics is recommended [5,6,7]. However, guidelines for treating PSI remain ambiguous due to variability in treatment duration and regional antibiotic resistance [8,9]. Furthermore, the destruction of spinal joints, such as the intervertebral disc and endplates, progressed by infection causes sustained back pain and disability even after the discontinuation of antibiotic treatment. Quality of life and the ability to return to work were significantly decreased in the patients with PSI regardless of treatment modality [10,11,12].

To the best of our knowledge, there are no sufficient data analyzing medical burden and functional status related with PSI. Treatment guidelines are also lacking, as mentioned above. In this study, we investigated the clinical factors associated with the length/cost of hospitalization for the medical burden and initial/6-month short form 36 (SF-36) for the functional status in the treatment of PSI, respectively.

## 2. Patients and Methods

### 2.1. Patients and Data Collection

This retrospective study involved a cohort of 156 patients with a non-postoperative PSI of the thoraco-lumbo-sacral area in a single tertiary university hospital from March 2015 to December 2021. Patients were excluded if they had any of the following: tuberculous spondylitis, tumors, accompanying bone infection at another site, trauma, pregnancy, less than six months of follow-up period, death, data loss, transferred from another hospital during the treatment, or age < 19 years. All clinical and radiological data were obtained and reviewed retrospectively from electronic charts under the approval of the Institutional Review Board.

### 2.2. Diagnosis of PSI

PSI was diagnosed based on clinical symptoms, laboratory data, and radiological findings. Clinical symptoms included fever and back pain with or without radiating pain. Elevated erythrocyte sedimentation rates (ESR, normal range: <20 mm/h), C-reactive protein (CRP, normal range: <0.5 mg/dL), or both, along with specific findings of magnetic resonance (MR) imaging of PSI, were important clues for diagnosis of PSI [13,14]. The PSI lesion can be presented as a single form or as a combination of vertebral osteomyelitis, discitis, septic arthritis of facet joint, and abscess formation in the epidural and/or paraspinal spaces in addition to the psoas and/or erector spine musculatures on MR imaging. All PSI with these various findings of MR imaging were included and their characteristics were analyzed. The extent of PSI was defined as the count of vertebral bodies containing a PSI lesion. For instance, there was spondylodiscitis of L3-4-5 with epidural abscess of L1-S1, which were defined as six levels.

### 2.3. Identification of Causative Bacteria and Treatment

In the patients diagnosed with PSI, a microbiological diagnosis was attempted through more than two sets of blood culture or tissue culture of the PSI lesion by computed tomography (CT)-guided needle biopsy or open surgical biopsy. PSI with appropriate clinical features and radiological findings, but with no identification of causative bacteria, were defined as culture-negative PSI; otherwise, they were defined as culture-positive PSI [15]. The choice of parenteral antibiotics was decided based on the clinical data and according to the recommendation of an infectious disease specialist. All patients underwent a minimum of six months of a follow-up period after completion of parenteral antibiotic treatment. Additional surgical treatment (decompression ± fixation) was added to antibiotic treatment in patients with neurologic deficits and/or sustained back pain caused by spinal instability.

### 2.4. Cure and Recurrence

Patients were considered as cured when they exhibited an absence of fever, improved clinical symptoms, and a sustained trend of normalized CRP after at least four weeks of parenteral antibiotic therapy. Recurrence was defined as a condition with CRP re-elevation, identification of newly developed, or aggravation of an existing PSI lesion on MR imaging, and with recurred or aggravated back pain with or without fever [16,17,18].

### 2.5. Medical Burden of the Treatment of PSI

The medical burden was defined as the length and cost of hospitalization. The cost of hospitalization consisted of the expenses related to medication, injection, consultation, care, procedure, laboratory or radiological examination, and hospital room charge. These also included the cost of various medical complications related with PSI treatment and control of previous accompanying diseases during the hospitalization, as well as the cost of treating PSI itself. We analyzed the effects of clinical factors associated with the length and cost of hospitalization, respectively.

### 2.6. Functional Status Related with PSI

The severity of low back pain was evaluated using SF-36 to present the PSI-related functional status. SF-36 consists of 36 items that result in a score from 0 to 100, where a higher score demonstrates a higher quality of life. A low back-specific version of SF-36 is commonly used to describe the status of chronic low back pain [19] and is also used for measuring morbidity and surgical outcomes [20]. SF-36 was applied initially and six months after completion of parenteral antibiotic treatment. We analyzed the effects of clinical factors associated with the initial and six-month SF-36s.

### 2.7. Statistical Analysis

Data distributions were firstly evaluated for normality by the Kolmogorov–Smirnov test. Student’s *t*-test and the Mann–Whitney U test were used to compare parametric and non-parametric continuous variables, respectively. Linear regression analysis was used to estimate the relationship between two continuous variables. Uni- and multi-variable logistic regression analyses were performed to test the effect of uni- or multi-independent variables for the binary dependent variable and to estimate odds ratio with 95% confidence intervals. Statistical analysis was carried out using SPSS version 27.0 software (SPSS Inc., Chicago, IL, USA), and a *p* value of < 0.05 was considered statistically significant.

## 3. Results

### 3.1. Clinical and Demographic Data

**Figure 1:**
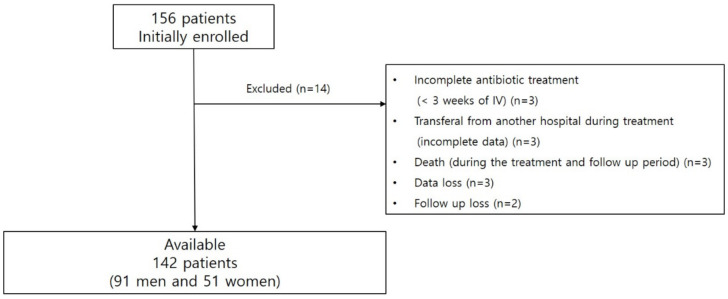
Flowchart of inclusion and exclusion process of PSI patients.

Among the 156 patients, 14 patients were excluded due to incomplete antibiotic treatment (n = 3), death (n = 3), data loss (n = 3), transferal from another hospital during antibiotic treatment (overall medical burden and initial functional status cannot be estimated, n = 3), and follow-up loss (n = 2). The final analyses were performed on the data from 142 patients (91 men and 51 women) with a mean age of 66.51 ± 11.76 (41–90) years (Figure 1). Diabetes mellitus was the most common underlying disease (28.2%, 40/142), and 61.3% (87/142) of patients had procedure-related PSI. A majority of patients (98.6%, 140/142) presented with back pain as the most common accompanied symptom, followed by leg radiculopathy (50.0%, 71/142), leg weakness (35.2, 50/142), fever (18.3%, 26/142), and bowel and bladder symptoms (2.8%, 4/142). The severity of comorbidity of the patients was shown as the Charlson’s comorbidity index (CCI) with 3.07 ± 1.77 (0–9). In the features of MR imaging, the extent of PSI lesion was 3.38 ± 1.69 (1–11) levels; and 66.2% (94/142) of epidural abscess, 77.5% (110/142) of paraspinal abscess, 34.5% (49/142) of psoas abscess, 75.4% (107/142) of discitis, and 33.8% (48/142) of back muscle abscess were noted. Bacteremia was accompanied in 22.5% (32/142) of the patients. The mean duration of susceptible parenteral antibiotics for PSI was 44.73 ± 18.00 (21–140) days, and additional surgeries including neural decompression with or without fixation were performed in 42.3% (60/142) of the patients.

The indices of the initial (at diagnosis) blood inflammatory markers including white blood cells (WBC), ESR, and CRP were 10,625 ± 4921 (3750–28,790), 71.23 ± 31.37 (2–120), and 9.57 ± 9.71 (0.03–38.00), respectively. The final indices of blood inflammatory markers at the time of stopping parenteral antibiotics were improved for WBC, ESR, and CRP as 5989 ± 1.98 (2340–15,020), 41.17 ± 26.63 (2–120), and 0.81 ± 1.46 (7–172). The length and cost of hospitalization were 55.56 ± 27.09 (7–172) days and 14,070.17 ± 9289.39 (1611.87–48,722.35) US dollars (mean exchange rate from March 2015 to December 2021; 1 US dollar [$] = 1146.44 Korea Won) during the treatment of PSI. In the functional status, there was a significant improvement of SF-36 from 34.20 ± 12.89 (3–57) at diagnosis to 49.12 ± 8.76 (26–60) at 6 months after the completion of the treatment (*p* < 0.05). All patients were followed up for a minimum of 6-months, and the mean follow-up period was 18.13 ± 14.99 months. The causative bacteria were identified in 43.7% (62/142) of the patients. *Staphylococcus aureus* was the most common causative bacteria (21.8%, 31/142) and methicillin resistance rate was noted in 38.7% (12/31) of the patients. The detailed data are presented in Table 1 and Table 2.

### 3.2. Clinical Factors Associated with Medical Burden

Among the clinical symptoms, leg weakness was related to an increase of length (*p* = 0.001) and cost (*p* = 0.000) of hospitalization. WBC, ESR, and CRP showed positive correlation with length (*r* = 0.223 and *p* = 0.008, *r* = 0.172 and *p* = 0.040, *r* = 0.300 and *p* = 0.000) and cost (*r* = 0.358 and *p* = 0.000, *r* = 0.224 and *p* = 0.007, *r* = 0.440 and *p* = 0.000) of hospitalization. Identification of causative bacteria and bacteremia related with an increase of the length (*p* = 0.028, *p* = 0.003) and cost (*p* = 0001, *p* = 0.001) of hospitalization. Additional surgical treatment and recurrence were related to the increase of the length (*p* = 0.000, *p* = 0.004) and cost (*p* = 0.000, *p* = 0.006) of hospitalization. The extent of lesion showed a positive correlation with the length (*p* = 0.001) and cost (*p* = 0.000) of hospitalization. Epidural and back muscle abscesses related to an increase of the length (*p* = 0.000, *p* = 0.009) and cost (*p* = 0000, *p* = 0.012) of hospitalization. Paraspinal abscess was only related to an increase of the cost (*p* = 0.030) of hospitalization. The length and cost of hospitalization showed a positive correlation with each other (*r* = 0.790, *p* = 0.000). The detailed data are presented in Table 3.

### 3.3. Clinical Factors Associated with Functional Status

Age was a statistically significant factor in functional status, as there was a negative correlation with both initial (*r* = −0.184 and *p* = 0.028) and 6-month SF-36 values (*r* = −0.219, *p* = 0.009). The male sex showed better SF-36 at the initial SF-36 (*p* = 0.024) as compared to females. Among the clinical symptoms, the presence of radiculopathy and weakness were related with poor initial (*p* = 0.006 and *p* = 0.003) and 6-month (*p* = 0.000 and *p* = 0.000) SF-36s. Bowel and bladder symptoms were related with poor initial SF-36 (*p* = 0.010). WBC and CRP showed negative correlations with both initial (*r* = −0.273 and *p* = 0.001, *r* = −0.313 and *p* = 0.000) and 6-month (*r* = −0.253 and *p* = 0.002, *r* = −0.240 and *p* = 0.004) SF-36s. CCI showed a negative correlation with both initial (*r* = −0.207 and *p* = 0.013) and 6-month (*r* = −0.204 and *p* = 0.004) SF-36s. Identification of causative bacteria and bacteremia were related with poor initial (*p* = 0.000, *p* = 0.000) and 6-month (*p* = 0.001, *p* = 0.037) SF-36s. The extent of lesion showed negative correlations with the both initial (*r* = −0.191 and *p* = 0.023) and 6-month (*r* = −0.168 and *p* = 0.046) SF-36s. Epidural, psoas, and back muscle abscesses were related with poor initial (*p* = 0.000, *p* = 0.003, *p* = 0.016) and 6-month (*p* = 0000, *p* = 0.000, *p* = 0.013) SF-36s. The length and cost of hospitalization showed negative correlations with both initial (*r* = −0.241 and *p* = 0.004, *r* = −0.343 and *p* = 0.000) and 6-month (*r* = −0.241 and *p* = 0.004, *r* = −0.321 and *p* = 0.000) SF-36s. The detailed data are presented in Table 4.

### 3.4. Clinical Factors Associated with Above-Average Medical Burden

Uni- and multi-variable logistic regression analyses were performed to investigate the effect of clinical factors for the above-average medical burden including length and cost of hospitalization. Leg weakness (OR 2.826), WBC (OR 1.101), CRP (OR 1.072), identification of causative bacteria (OR 2.187), bacteremia (OR 4.455), additional surgical treatment (OR 5.333), recurrence (OR 8.600), extent of lesion (OR 1.590), epidural abscess (OR 4.792), and back muscle abscess (OR 2.436) in univariable analysis; and procedure-related (OR 2.702), CRP (OR 1.062), bacteremia (OR 4.966), additional surgical treatment (OR 6.524), recurrence (OR 12.453), and paraspinal abscess (OR 5.965) in multivariable analysis; were statistically significant factors for the above-average length of hospitalization. Female (OR 4.438), fever (OR 2.395), leg weakness (OR 2.750), WBC (OR 1.110), CRP (OR 1.075), identification of causative bacteria (OR 3.200), bacteremia (OR 4.865), additional surgical treatment (OR 5.353), recurrence (OR 9.209), extent of lesion (OR 1.665), epidural abscess (OR 4.400), and psoas abscess (OR 2.229) in univariable analysis; and CRP (OR 1.071), bacteremia (OR 4.647), additional surgical treatment (OR 6.737), recurrence (OR 22.543), extent of lesion (OR 1.431) in multivariable analysis; were statistically significant factors for the above-average cost of hospitalization. The detailed data are presented in Table 5.

### 3.5. Clinical Factors Associated with Below-Average Functional Status

Uni- and multi-variable logistic regression analyses were performed to investigate the effect of clinical factors for the below-average functional status including initial and 6-month SF-36s. Leg radiculopathy (OR 2.902), leg weakness (OR 17.503), WBC (OR 1.159), CRP (OR 1.060), CCI (OR 1.283), identification of causative bacteria (OR 4.764), bacteremia (OR 5.042), extent of lesion (OR 1.356), epidural abscess (OR 2.692), and back muscle abscess (OR 2.377) in univariable analysis; and leg weakness (OR 15.966), WBC (OR 1.116), CCI (OR 1.283), and identification of causative bacteria (OR 4.764) in multivariable analysis; were statistically significant factors for the below-average initial SF-36. Female (OR 2.287), leg radiculopathy (OR 3.152), leg weakness (OR 6.375), WBC (OR 1.093), CRP (OR 1.040), identification of causative bacteria (OR 2.077), epidural abscess (OR 2.755), psoas abscess (OR 2.577), and back muscle abscess (OR 2.106) in univariable analysis; leg weakness (OR 7.975) and WBC (OR 1.094) in multivariable analysis; and were statistically significant factors for the below-average 6-month SF-36. The detailed data are presented in Table 6.

## 4. Discussion

In this study, we analyzed the clinical factors associated with the medical burden and functional ability of PSI. Elevation of initial blood inflammatory markers (WBC and CRP), presence of weakness, wide extent of the PSI lesion, combined epidural or back muscle abscess, identification of the causative bacteria, and the presence of bacteremia were statistically significant factors associated with increasing both the medical burden and functional disability. In the literature, old age and more comorbidities are associated with higher mortality rates, more adverse events, and prolonged hospitalization in the treatment of PSI [10,21]. Contrary to the common expectation, age and comorbidity showed no statistical significance in the medical burden presented as the length and cost of hospitalization; however, they were one of the major factors related to the initial and 6-month functional disability in this study. Our data suggest that functional disability is related to functional ability before PSI, which is correlative with age, comorbidity, and degenerative spinal problems presented as radiculopathy. Based on these results, we expect that the medical burden and functional disability mainly depend on the severity of infectious status and involvement of PSI lesion. ESR and CRP levels correlate with the presence of an inflammatory response; higher ESR and CRP may be more likely to occur in more infectious conditions [22]. In this study, ESR was a significant factor that increased medical burden such as WBC and CRP, but was not related to function disability. In particular, identification of the causative bacteria and bacteremia were associated with increased medical burden and functional disability than otherwise, which may be associated with increased blood inflammatory markers, extent of the lesion, and presence of abscess. A previous study in Korea showed that a spinal epidural abscess with bacteremia required a longer duration (>8 weeks) of antibiotic treatment [23].

Previous studies have presented guidelines for the treatment of PSI. However, there are still no definite guidelines for the use of antibiotics due to the differences in the causative pathogen, antibiotic resistance rate, and variations in regional medical systems. The guideline of Infectious Diseases Society of America suggests the use of parenteral antibiotics or oral antibiotics with a high bioavailability for more than six weeks. Although some authors in regions with a low incidence of antibiotic resistance recommend changing to oral antibiotics after relatively short-term parenteral antibiotics less than two weeks, this recommendation should be cautiously applied in regions with a high rate of antibiotic resistance. In Korea, the commonly isolated bacteria of PSI are *Staphylococcus aureus* (58.8%), *Streptococcus* species (11.3%), *Escherichia coli* (11.0%), and the incidence of methicillin-resistant *Staphylococcus aureus* is noted as 43.3% [24]. Additionally, there is a high incidence of antibiotic resistance of *Staphylococcus* to methicillin (80%), K. pneumoniae to ceftazidime (25~35%) and quinolone (30%), and *Pseudomonas aeruginosa* to ceftazidime (21%) and imipenem (17%) in nosocomial infection [25,26,27]. Consequently, the final effective antibiotics are usually focused on methicillin-resistant *Staphylococcus aureus* and multi-drug resistant gram-negative pathogens in patients with a high risk or a poor response for the initial empirical antibiotics regardless of the identification of the causative pathogen, which can result in the long-term use of parenteral antibiotics and hospitalization for PSI in Korea.

The medical burden depends on the length and cost of hospitalization. The cost of hospitalization consists of expenses involved in the room charge, prescription, examination, and nursing mainly incurred during the peri-antibiotic therapy period. Generally, after four weeks of hospitalization with antibiotic therapy, overall conditions such as back pain and fever improve [28]. Considering that the main cause of hospitalization is for the administration of parenteral antibiotics due to a higher rate of antibiotic resistance, it is required to consider using oral antibiotics with high bioavailability in order to decrease medical burden and use medical resources efficiently. Several studies suggest that switching antibiotic therapy from parenteral to oral administration for patients with PSI has several advantages, including reduced length of hospitalization. Li et al. [29] reported in their open-labeled randomized trial reported that oral antibiotics might be non-inferior to parenteral therapy for complex orthopedic infection. Flury et al. [30] reported that switching oral antibiotics after two weeks of parenteral antibiotics may be safe for primary bacterial vertebral osteomyelitis. However, these studies are limited by the fact that their analyses did not perform any sub-group analysis depending on the pathogens and their antimicrobial susceptibility. The risk of newly increased antibiotic resistance rate means that the wide use of high bioavailable oral antibiotics should be considered.

In our results, recurrence (7.7%, 11/142) was identified as a significant factor for increasing medical burden, unlike functional disability. The recurrence rates in the previous studies vary widely. One study in the US showed a recurrence rate of approximately 14% (35/253) in which 90% of them received more than four weeks of antibiotic therapy [31]. In another study in Korea, there was 9.9% (31/319) of recurrence after 49 days of parenteral antibiotics therapy for hematogenous vertebral osteomyelitis [24]. According to a recent study in Denmark, the recurrence rate was 13% (12/93) after 87 days of treatment with a mean of 34 days of parenteral and 52 days of oral antibiotic therapy [32]. However, there was no significant difference in recurrence rate between the patients who received less than six weeks of antibiotic therapy (with 20 ± 16 days of parenteral antibiotic therapy) and other patients who underwent more than six weeks (with 35 ± 16 days of parenteral antibiotic therapy) [33]. Unfortunately, there is still no definite method to evaluate and achieve an accurate therapeutic response to avoid recurrence. Although the CRP is still considered as the most useful and widely used method for evaluating therapeutic response, it has low specificity and vulnerability to other general conditions [34,35]. Accurate determination of therapeutic response with low recurrence is an important issue for the improvement of the overall prognosis of PSI, which is associated with medical burden. Recently, positron emission tomography was introduced as a new modality to evaluate therapeutic response in PSI, and further studies are needed to apply this as real-world evidence in terms of value of evaluating therapeutic response and cost-effectiveness [14,36].

There are several limitations in our study. First, PSI is being treated in various settings such as a department of infectious medicine, orthopedics, and spinal neurosurgery. The participants in this study were patients with PSI treated in a spinal neurosurgery department. The relatively low recurrence (7.7%, 11/142) and death rates (1.9%, 3/156) could be attributed to our participants having relatively better general conditions compared to those who are treated in infectious medicine departments. However, the mean CCI of the participants of this study was 3.07 ± 1.77, which is higher than the mean CCI (2.42 ± 2.12 for a group without recurrence, 2.28 ± 2.22 for a group with recurrence) in a nationwide cohort study of 2148 patients [37]. We presume that long-term parenteral antibiotic therapy for PSI is appropriate for low rates of recurrence and death in regions with a higher antibiotic resistance. Moreover, our cohort only included non-postoperatively developed PSI, which may also result in the low recurrence and death rate. Second, this is a single center study including a relatively small population under a retrospective study design. However, a nationwide cohort study including 7305 pyogenic vertebral osteomyelitis patients in Korea reported individual medical costs of $10,049 ± 94 in 2007, and $16,672 ± 17,729 in 2016 [38]. Our study data show $14,070.17 ± 9289.39, which is a similar figure considering the gap in which the participants were enrolled. Furthermore, our study also presented the clinical factors associated with the medical burden and functional disability of PSI, which can be clinically useful data in the real world. Third, the study population does not include post-operative PSI; therefore, the results do not represent all types of PSIs. Interestingly, there was no significant difference in the medical burden and functional disability between spontaneous and procedure-related (non-surgical) PSI groups in the *t*-test. However, the procedure-related factor showed statistical significance for the length of hospitalization in the multivariable analysis (OR 2.702). Some studies reported that lumbar epidural injection is considered to be a safe procedure and only 0.001–0.1% of spine infections caused by it require hospitalization [39,40,41]. However, little is known about the epidemiology of the difference in severity, recurrence rate, and functional outcome between spontaneous and procedure-related PSIs.

## 5. Conclusions

This study, for the first time, reports the PSI-associated medical burden and functional status. Recurrence and leg weakness were identified as the most important clinical factors for medical burden and 6-month functional status in PSI, respectively. We think that it is necessary to actively suppress recurrence and manage neurological deficits for decreasing medical burden and achieving favorable functional outcome in the treatment of PSI.

## Figures and Tables

**Table 1:** Clinical and demographic data. CCI, Charlson’s comorbidity index; MR, magnetic resonance; WBC, white blood cell; ESR, erythrocyte sedimentation ratio (normal range < 20 mm/h); CRP, C-reactive protein (normal range < 0.5 mg/dL); $, US dollar (mean exchange rate from March 2015 to December 2021; 1 US dollar = 1146.44 Korea Won); SF-36, short form 36; Data are presented as the mean ± standard deviation or frequency (%).

Factors	Values (%)
Age, years	66.51 ± 11.76 (41–90)
Sex, male:female	91:51
Underlying diseases	
Diabetes mellitus	40 (28.2)
Rheumatic disease	7 (4.9)
Liver disease	3 (2.1)
Chronic kidney disease	3 (2.1)
Procedure-related (non-surgical)	87 (61.3)
Epidural injection	66 (75.9)
Acupuncture	13 (14.9)
Epidural injection & acupuncture	8 (9.2)
Clinical symptoms	
Fever, ≥37.3 °C	26 (18.3)
Back pain	140 (98.6)
Leg radiculopathy	71 (50.0)
Leg weakness	50 (35.2)
Bowel and bladder symptoms	4 (2.8)
CCI	3.07 ± 1.77 (0–9)
Features of MR imaging	
Extent of lesion, levels	3.38 ± 1.69 (1–11)
Epidural abscess	94 (66.2)
Paraspinal abscess	110 (77.5)
Psoas abscess	49 (34.5)
Discitis	107 (75.4)
Back muscle abscess	48 (33.8)
Initial blood inflammatory markers	
WBC, count	10,625 ± 4921 (3750–28,790)
ESR, mm/h	71.23 ± 31.37 (2–120)
CRP, mg/dL	9.57 ± 9.71 (0.03–38.00)
Last blood inflammatory markers (at completion of antibiotic therapy)	
WBC, count	5989 ± 1.98 (2340–15,020)
ESR, mm/h	41.17 ± 26.63 (2–120)
CRP, mg/dL	0.81 ± 1.46 (0.02–13.36)
Duration of parenteral antibiotics, days	44.73 ± 18.00 (21–140)
Additional surgical treatment	60 (42.3)
Length of hospitalization, days	55.56 ± 27.09 (7–172)
Cost of hospitalization, $	14,070.17 ± 9289.39 (1611.87–48,722.35)
Initial SF-36 (at diagnosis)	34.20 ± 12.89 (3–57)
6-month SF-36 (after completion of antibiotic therapy)	49.12 ± 8.76 (26–60)
Follow up period (after completion of antibiotic therapy), months	18.13 ± 14.99
Recurrence	11 (7.7)

**Table 2:** Microbiologic findings.

Causative Pathogens	Values (%)
Culture-positive	62 (43.7)
Gram-positive bacteria	
Staphylococcus aureus	31 (21.8)
Methicillin-sensitive	19
Methicillin-resistant	12
Coagulase-negative staphylococci	4 (2.8)
Streptococcus species	9 (6.3)
Enterococcus species	4 (2.8)
Gram-negative bacteria	
Acinetobacter	3 (2.1)
Achrombacter	1 (0.7)
Enterobacter	1 (0.7)
Klebsiella	4 (2.8)
Escherichia coli	4 (2.8)
Pseudomonas	1 (0.7)
Culture-negative	80 (56.3)

**Table 3:** Clinical factors associated with medical burden. WBC, white blood cell; ESR, erythrocyte sedimentation ratio (normal range < 20 mm/h); CRP, C-reactive protein (normal range < 0.5 mg/dL); CCI, Charlson comorbidity index; MR, magnetic resonance, $, US dollar (mean exchange rate from March 2015 to December 2021; 1 US dollar = 1146.44 Korea Won); *, *p* < 0.05.

Factors	Length of Hospitalization (Days)		Cost of Hospitalization ($)	
	Values	*p*	Values	*p*
Age, years	*r* = 0.014	0.869	*r* = 0.021	0.805
Sex				
Male	53.67 ± 27.54	0.267	12,926.69 ± 8324.47	0.068
Female	58.94 ± 26.18		16,110.50 ± 10,582.32	
Procedure−related				
(−)	53.71 ± 25.48	0.519	14,543.60 ± 9631.17	0.631
(+)	56.74 ± 28.14		13,770.87 ± 9110.53	
Clinical symptoms				
Fever, >37.3 °C				
(−)	54.77 ± 27.70	0.461	13,302.40 ± 9021.03	0.037
(+)	59.12 ± 24.34		17,495.58 ± 9869.66	
Back pain				
(−)	30.00 ± 5.66	0.180	5306.99 ± 370.78	0.180
(+)	55.93 ± 27.10		14,195.36 ± 9295.84	
Leg radiculopathy				
(−)	53.49 ± 26.15	0.365	12,737.38 ± 8208.99	0.087
(+)	57.63 ± 28.02		10,140.39 ± 1203.44	
Leg weakness				
(−)	50.30 ± 24.63	0.001 *	11,886.06 ± 7774.35	0.000 *
(+)	65.24 ± 28.94		18,088.94 ± 10,518.01	
Bowel and bladder symptoms				
(−)	55.38 ± 27.17	0.631	14,012.61 ± 9288.03	0.666
(+)	62.00 ± 26.57		16,055.98 ± 10,526.08	
Initial blood inflammatory markers				
WBC, count	*r* = 0.223	0.008 *	*r* = 0.358	0.000 *
ESR, mm/h	*r* = 0.172	0.040 *	*r* = 0.224	0.007 *
CRP, mg/dL	*r* = 0.300	0.000 *	*r* = 0.440	0.000 *
CCI	*r* = 0.080	0.344	*r* = −0.002	0.982
Identification of causative bacteria				
(−)	51.01 ± 22.86	0.028 *	11,563.03 ± 6371.57	0.001 *
(+)	61.44 ± 30.93		17,305.18 ± 11,314.02	
Bacteremia				
(−)	52.02 ± 25.78	0.003 *	12,410.88 ± 7909.22	0.001 *
(+)	67.75 ± 28.31		19,773.99 ± 11,366.02	
Additional surgical treatment				
(−)	46.40 ± 18.58	0.000 *	10,5333.59 ± 6647.80	0.000 *
(+)	68.08 ± 31.68		18,903.50 ± 10,218.26	
Recurrence				
(−)	52.02 ± 22.42	0.004 *	13,093.90 ± 8370.68	0.006 *
(+)	97.82 ± 41.02		25,696.66 ± 12,027.55	
Features of MR imaging at diagnosis				
Extent of lesion, levels	*r* = 0.275	0.001 *	*r* = 0.406	0.000 *
Epidural abscess				
(−)	44.48 ± 22.00	0.000 *	9175.41 ± 5855.07	0.000 *
(+)	61.22 ± 27.79		16,569.62 ± 9737.84	
Paraspinal abscess				
(−)	51.34 ± 26.09	0.318	11,576.66 ± 6252.27	0.030 *
(+)	56.79 ± 27.36		14,795.56 ± 9907.19	
Psoas abscess				
(−)	53.97 ± 27.13	0.335	13,010.61 ± 8335.86	0.061
(+)	58.59 ± 27.03		16,081.18 ± 10,677.02	
Discitis				
(−)	52.63 ± 22.17	0.462	13,523.31 ± 10,236.22	0.690
(+)	56.52 ± 28.54		14,249.05 ± 9002.55	
Back muscle abscess				
(−)	50.95 ± 23.55	0.009 *	12,554.67 ± 8229.47	0.012 *
(+)	64.60 ± 31.27		17,038.03 ± 10,549.01	
Length of hospitalization, days	-	-	*r* = 0.790	0.000 *
Cost of hospitalization, $	*r* = 0.790	0.000 *	-	-

**Table 4:** Clinical factors associated with functional status. SF-36, short form 36; WBC, white blood cell; ESR, erythrocyte sedimentation ratio (normal range < 20 mm/h); CRP, C-reactive protein (normal range < 0.5 mg/dL); CCI, Charlson comorbidity index; MR, magnetic resonance, *, *p* < 0.05.

Factors	Initial SF-36		6-Month SF-36	
	Values	*p*	Values	*p*
Age, years	*r* = −0.184	0.028 *	*r* = −0.219	0.009 *
Sex				
Male	36.02 ± 12.17	0.024 *	50.18 ± 8.18	0.055
Female	30.94 ± 13.60		47.24 ± 9.50	
Procedure−related				
(−)	31.65 ± 14.68	0.078	47.33 ± 9.29	0.052
(+)	35.80 ± 11.41		50.25 ± 8.27	
Clinical symptoms				
Fever, >37.3 °C				
(−)	34.63 ± 12.74	0.401	49.44 ± 8.18	0.453
(+)	32.27 ± 13.64		47.69 ± 11.06	
Back pain				
(−)	38.50 ± 7.78	0.636	54.50 ± 6.36	0.384
(+)	34.14 ± 12.95		49.04 ± 8.78	
Leg radiculopathy				
(−)	37.17 ± 11.26	0.006 *	51.25 ± 6.84	0.003 *
(+)	31.23 ± 13.78		46.99 ± 9.93	
Leg weakness				
(−)	40.70 ± 7.93	0.000 *	52.71 ± 5.54	0.000 *
(+)	22.24 ± 11.66		42.52 ± 9.76	
Bowel and bladder symptoms				
(−)	34.67 ± 12.64	0.010 *	49.32 ± 8.57	0.112
(+)	18.00 ± 12.25		42.25 ± 13.77	
Initial blood inflammatory markers				
WBC, count	*r* = −0.273	0.001 *	*r* = −0.253	0.002 *
ESR, mm/h	*r* = −0.076	0.369	*r* = −0.132	0.118
CRP, mg/dL	*r* = −0.313	0.000 *	*r* = −0.240	0.004 *
CCI	*r* = −0.207	0.013 *	*r* = −0.204	0.004 *
Identification of causative bacteria				
(−)	37.83 ± 10.68	0.000 *	51.38 ± 6.10	0.001 *
(+)	29.52 ± 14.03		46.21 ± 10.67	
Bacteremia				
(−)	36.67 ± 11.32	0.000 *	50.21 ± 7.26	0.037 *
(+)	25.69 ± 14.49		45.38 ± 12.04	
Additional surgical treatment				
(−)	35.70 ± 12.62	0.106	49.70 ± 8.18	0.362
(+)	32.15 ± 13.08		48.33 ± 9.51	
Recurrence				
(−)	34.37 ± 12.95	0.574	49.31 ± 8.74	0.385
(+)	32.09 ± 12.48		46.91 ± 9.15	
Features of MR imaging at diagnosis				
Extent of lesion, levels	*r* = −0.191	0.023 *	*r* = −0.168	0.046 *
Epidural abscess				
(−)	38.71 ± 8.56	0.000 *	52.10 ± 4.66	0.000 *
(+)	31.89 ± 14.11		47.60 ± 9.92	
Paraspinal abscess				
(−)	36.53 ± 9.92	0.172	51.13 ± 6.27	0.072
(+)	33.52 ± 13.60		48.54 ± 9.30	
Psoas abscess				
(−)	36.78 ± 10.96	0.003 *	51.59 ± 6.47	0.000 *
(+)	29.29 ± 14.84		44.43 ± 10.53	
Discitis				
(−)	35.43 ± 12.30	0.517	51.17 ± 8.09	0.111
(+)	33.79 ± 13.11		48.45 ± 8.90	
Back muscle abscess				
(−)	36.04 ± 12.91	0.016 *	50.50 ± 8.05	0.013 *
(+)	30.58 ± 12.18		46.42 ± 9.53	
Length of hospitalization, days	*r* = −0.241	0.004 *	*r* = −0.241	0.004 *
Cost of hospitalization, $	*r* = −0.343	0.000 *	*r* = −0.321	0.000 *

**Table 5:** Logistic regression analysis of clinical factors associated with above-average medical burden. WBC, white blood cell; ESR, erythrocyte sedimentation ratio (normal range < 20 mm/h); CRP, C-reactive protein (normal range < 0.5 mg/dL); CCI, Charlson comorbidity index; *, *p* < 0.05.

	Length of Hospitalization (Days)						Cost of Hospitalization ($)					
Factors	Univariable Analysis			Multivariable Analysis			Univariable Analysis			Multivariable Analysis		
	OR	95% CI	*p*	OR	95% CI	*p*	OR	95% CI	*p*	OR	95% CI	*p*
Age, years	0.993	0.965–1.022	0.633				1.013	0.984–1.044	0.384			
Sex (female)	1.807	0.895–3.648	0.099				2.279	1.121–4.635	0.023 *	4.438	1.524–2.920	0.006 *
Procedure-related	1.451	0.716–2.941	0.302	2.702	1.072–6.810	0.035 *	0.896	0.446–1.803	0.759			
Fever, >37.3 °C	1.829	0.776–4.314	0.168				2.395	1.010–5.676	0.047 *			
Leg radiculopathy	1.828	0.920–3.632	0.085				1.630	0.818–3.246	0.165			
Leg weakness	2.826	1.383–5.775	0.004 *				2.750	1.343–.5.631	0.006 *			
WBC	1.101	1.024–1.184	0.009 *				1.110	1.032–1.194	0.005 *			
ESR, mm/h	1.011	1.000–1.022	0.061				1.008	0.997–1.020	0.141			
CRP, mg/dL	1.072	1.031–1.113	<0.001 *	1.062	1.010–1.118	0.019 *	1.075	1.035–1.118	<0.001 *	1.071	1.010–1.135	0.022 *
CCI	1.012	0.835–1.226	0.906				1.064	0.877–1.291	0.531			
Identification of causative bacteria	2.187	1.097–4.363	0.026 *				3.200	1.573–6.509	0.001 *			
Bacteremia	4.455	1.931–10.274	<0.001 *	4.966	1.706–14.453	0.003 *	4.865	2.101–11.263	<0.001 *	4.647	1.411–15.299	0.012 *
Additional surgical treatment	5.333	2.557–11.122	<0.001 *	6.524	2.570–16.562	<0.001 *	5.353	2.552–11.226	<0.001 *	6.737	2.276–19.942	<0.001 *
Recurrence	8.600	1.782–41.504	0.007 *	12.453	2.139–72.509	0.005 *	9.209	1.906–44.487	0.006 *	22.543	3.682–138.013	<0.001 *
Extent of lesion, levels	1.590	1.242–2.035	<0.001 *				1.665	1.291–2.147	<0.001 *	1.431	1.010–2.028	0.044 *
Epidural abscess	4.792	2.027–11.325	<0.001 *				4.400	1.861–10.402	<0.001 *			
Paraspinal abscess	1.769	0.749–4.178	0.193	5.965	1.701–20.923	0.005 *	2.000	0.824–4.853	0.125			
Psoas abscess	1.553	0.766–3.150	0.222				2.229	1.091–4.552	0.028 *			
Discitis	1.051	0.478–2.313	0.901				1.144	0.514–2.547	0.741			
Back muscle abscess	2.436	1.191–4.984	0.015 *				1.805	0.884–3.688	0.105			

**Table 6:** Logistic regression analysis of clinical factors associated with below-average functional ability. SF-36, short form 36; WBC, white blood cell; ESR, erythrocyte sedimentation ratio (normal range < 20 mm/h); CRP, C-reactive protein (normal range < 0.5 mg/dL); CCI, Charlson comorbidity index; *, *p* < 0.05, ^#^, only included in the analyses of 6-month SF-36.

	Initial SF-36						6-month SF-36					
Factors	Univariable Analysis			Multivariable Analysis			Univariable Analysis			Multivariable Analysis		
	OR	95% CI	*p*	OR	95% CI	*p*	OR	95% CI	*p*	OR	95% CI	*p*
Age, years	1.025	0.995–1.055	0.099				1.023	0.993–1.053	0.135			
Sex (female)	1.977	0.985–3.967	0.055				2.287	1.133–4.618	0.021 *			
Procedure-related	0.561	0.283–1.113	0.098				0.546	0.274–1.087	0.085			
Fever, >37.3 °C	1.214	0.516–2.855	0.656				1.352	0.574–3.185	0.490			
Leg radiculopathy	2.902	1.453–5.795	0.003 *				3.152	1.561–6.362	0.001 *			
Leg weakness	17.503	7.256–42.220	<0.001 *	15.966	5.967–42.724	<0.001 *	6.375	2.985–13.615	<0.001 *	7.975	3.307–19.232	<0.001 *
WBC	1.159	1.068–1.257	<0.001 *	1.116	1.007–1.237	0.036 *	1.093	1.017–1.174	0.015 *	1.094	1.006–1.190	0.035 *
ESR, mm/h	1.002	0.992–1.013	0.668				1.007	0.996–1.018	0.202			
CRP, mg/dL	1.060	1.021–1.100	0.002 *				1.040	1.004–1.077	0.029 *			
CCI	1.283	1.049–1.569	0.015 *	1.485	1.130–1.952	0.005 *	1.142	0.942–1.385	0.177			
Identification of causative bacteria	4.764	2.327–9.754	<0.001 *	2.913	1.087–7.803	0.033 *	2.077	1.049–4.113	0.036 *			
Bacteremia	5.042	2.120–11.989	<0.001 *				1.429	0.646–3.159	0.378			
Additional surgical treatment^#^	-	–	–				1.418	0.719–2.796	0.313			
Recurrence ^#^	-	–	–				2.835	0.790–10.177	0.110			
Extent of lesion, levels	1.356	1.087–1.692	0.007 *				1.111	0.911–1.355	0.299			
Epidural abscess	2.692	1.267–5.723	0.010 *				2.755	1.278–5.941	0.010 *			
Paraspinal abscess	1.290	0.575–2.897	0.537				1.154	0.513–2.595	0.729			
Psoas abscess	1.962	0.972–3.958	0.060				2.577	1.265–5.249	0.009 *			
Discitis	1.131	0.520–2.460	0.756				1.391	0.627–3.085	0.417			
Back muscle abscess	2.377	1.168–4.836	0.017 *				2.106	1.036–4.279	0.040 *			

## Data Availability

The datasets acquired and analyzed during the current study are available from the corresponding author on the reasonable request.
